# Protein arginine methyltransferases in protozoan parasites: a new path for antiparasitic chemotherapy?

**DOI:** 10.15698/mic2026.02.869

**Published:** 2026-02-12

**Authors:** Gustavo D. Campagnaro, Sébastien Pomel

**Affiliations:** 1 Department of Cell and Molecular Biology, Ribeirão Preto Medical School, University of São Paulo, Ribeirão Preto, 14049-900, São Paulo, Brazil; 2 Université Paris-Saclay, CNRS BioCIS, 91400, Orsay, France

**Keywords:** arginine methyltransferase, protozoan parasites, kinetoplastid, apicomplexan, amoeba, antiparasitic chemotherapy

## Abstract

Protein arginine methyltransferases (PRMTs) catalyse the transference of methyl groups from S-adenosylmethionine to arginine residues in substrate proteins, a post-translational modification widespread among eukaryotes. The change in size and hydrophobicity of the methylated arginine residue impacts on how a protein interacts with other macromolecules and affects several cellular processes, including intracellular signaling, DNA replication and repair, and control of gene expression. As a result, PRMTs became attractive targets for chemotherapy, and several PRMT inhibitors are going through clinical trials for cancer treatment. In protozoan parasites, PRMTs play fundamental roles during development, stage differentiation and infection processes. We here review the activity and the relevance of PRMTs for the survival of pathogenic kinetoplastids, apicomplexans and amoebas, highlight differences observed between PRMTs expressed in these organisms and their mammalian orthologues, and suggest that these enzymes can be exploited to combat parasitic infections. We propose that the arsenal of inhibitors developed to target mammalian PRMTs could be reassigned to allow the identification of new scaffolds to be explored as antiparasitic agents, either as sole chemotherapy or by improving the effectiveness of current antiparasitic drugs.

## INTRODUCTION

Protozoan parasites often display complex life cycles encompassing several biological forms within many potential hosts. The transition from one biological form to another is triggered by environmental cues that culminate in an extensive metabolic rearrangement that requires fine-tuned gene expression control at various levels, from modifying the accessibility of chromatin regions to the sequestration or degradation of RNAs.

Alterations in chromatin accessibility are largely mediated by the set of dynamic post-translational modifications (PTMs) that occur at histone tails, including acylations, methylation, phosphorylation, and ubiquitination, among others, and that modify the dynamics of histone-DNA interaction to allow (or block) the access of repair, transcription and replication machineries to the DNA double-strand 1. In protozoans, a series of histone variants carrying divergent PTMs have been reported, and their function on DNA replication, transcription initiation and termination, and DNA damage signaling have started being dissected in the last decades 2–5.

Moreover, RNA-binding proteins (RBPs) play an essential role in the control of gene expression by altering transcript fate, *i.e.* its processing and rate of nuclear export and translation, as well as RNA storage and degradation, which is particularly important for organisms as trypanosomatids that do not possess a strict control of transcription 6. PTMs present in RBPs can affect their stability, intracellular localization and interaction with other macromolecules, thus altering the dynamics of their interaction with RNA and the formation of multiprotein complexes 7.

**Figure 1:**
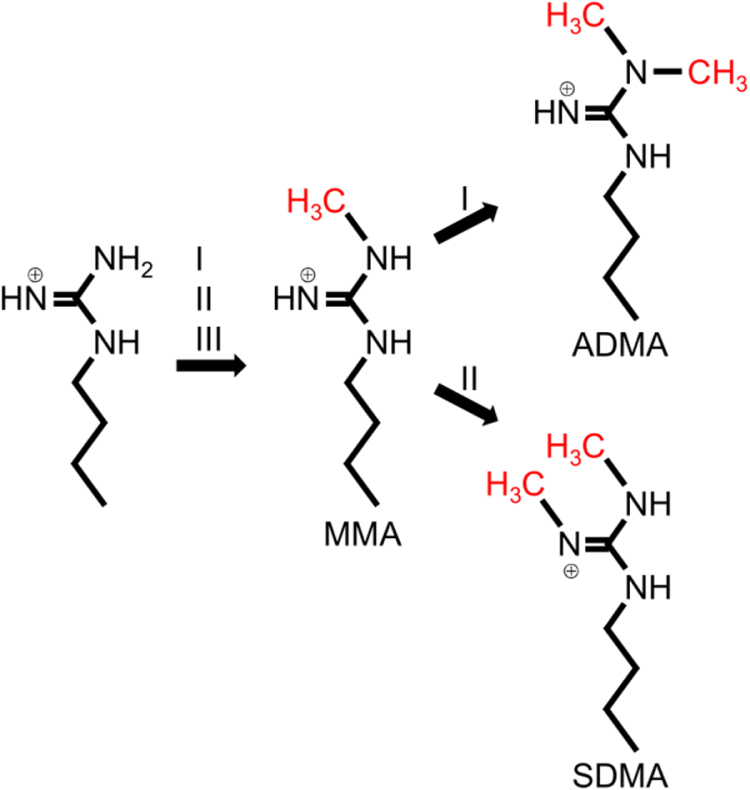
Schematic representation of the three major types of arginine methylation catalysed by PRMTs. While type I, II and III PRMTs can catalyse the formation of monomethylarginine (MMA), enzymes of types I and II catalyse the addition of a second methyl group to the same residue, originating asymmetrical (ADMA) and symmetrical (SDMA) dimethylarginines.

Among the group of enzymes that modify both histones and RBPs, we highlight the arginine methyltransferases (PRMTs), the enzymes which transfer a methyl group from S-adenosylmethionine to the terminal guanidino nitrogen of arginine residues augmenting their size and hydrophobicity, while reducing its ability to form hydrogen bonds, but without modifying its charge 8. These enzymes are categorized within four types, of which types I, II and III are widely distributed among eukaryotes, whereas type IV PRMTs are only present in fungi and probably in plants. Type I, II and III PRMTs transfer the methyl group from S-adenosylmethionine (SAM) to one of the terminal ( ω ) nitrogen atoms of arginine to generate ω -monomethylarginine (MMA). Type I PRMTs can add a second methyl group to the same nitrogen to form asymmetric ω -dimethylarginine (ADMA) while type II PRMTs modify the other ω -nitrogen to generate symmetric ω -dimethylarginine (SDMA) (Figure 1). Type IV PRMTs, on the other hand, monomethylate the internal ( δ ) guanidino nitrogen 9.

The PRMTs are constituted of six conserved domains: (1) motif I (VLD/VGxGxG), (2) motif post-I (V/I-X-G/A-X-D/E), (3) motif II (E/K/VDII), (4) double-E loop (SExMGxxLxxExM), (5) motif III (LK/xxGxxxP) and (6) the THW loop (THWxQ), which are responsible for substrate binding and stabilization. Moreover, type I PRMTs possess a generally conserved YFxxY motif in their N-terminal portion; the equivalent motif in type II PRMTs is PLxxN 10.

The altered expression of PRMTs has been correlated with cancer development and progression, which in turn propelled the efforts to develop effective and selective PRMT inhibitors, particularly of type I and type II PRMTs, and most notably of PRMT5, some of which are already undergoing clinical trials, either alone or in combination with traditional oncodrugs 11–14. As the biochemical, physiological and structural studies on the human PRMTs advance, the development of more specific and active compounds will become easier. Nonetheless, a large number of molecules will unequivocally fail preclinical or clinical tests 15, 16, making it a golden pot of potential antiparasitic agents.

Although protozoan PRMTs display the same conserved motifs as mammalian PRMTs, the recent disclosure of their functionality and elucidation of their structures revealed certain peculiarities that may allow the repurposing of already tested human PRMT inhibitors and serve as scaffold for the development of specific chemotherapies. Moreover, the recent discovery on the impact of arginine methylation in core cellular and biological aspects of pathogenic protists allows the establishment of these enzymes as attractive drug targets against these pathogens. Thus, we here review the relevance of PRMT activities for the survival of protozoan parasites, how PRMT inhibitors can be used as antiparasitic agents, and propose a reflection on how the large arsenal of molecules designed to inhibit human PRMTs could be resignified to combat pathogenic protozoa.

## MAMMALIAN AND PROTOZOAN PRMTs

The nine PRMTs identified in humans are responsible for methylating a vast number of proteins, especially targeting those related to RNA processing and translation 17. And although showing a great substrate overlapping, these nine proteins display different functional activity, target selectivity and tissue and intracellular distribution. For instance, while the human PRMTs 1, 3, 5, and 8 display a preference for methylating arginine residues contained in arginine- and glycine-rich motifs, PRMT2 methylates proteins with proline-rich motifs as well as serine- or arginine- rich motifs, and PRMT4 (also known as coactivator associated arginine methyltransferase 1 (CARM1)) exhibits a distinct preference for methylating arginine residues contained in motifs rich in proline, glycine and methionine 9, 18. Protozoa, on the other hand, have between three and six genes (although many still require functional and biological validation) encoding for PRMTs: of these, all protozoa express at least two type I and one type II PRMTs, which is (more than) enough to cover the whole spectrum of methylarginine variants found in these eukaryotes. The protozoan PRMTs are named according to their corresponding human orthologue.

## KINETOPLASTID PRMTs

Kinetoplastids are mainly known for harbouring the causative agents of three of the 20 neglected diseases listed by the World Health Organization 19: Chagas’ disease (*Trypanosoma cruzi*), leishmaniases (*Leishmania* spp.) and sleeping sickness (*Trypanosoma brucei*), all belonging to the family Trypanosomatidae, which also harbours important veterinary pathogens, such as *Trypanosoma vivax*, *Trypanosoma evansi* and *Trypanosoma congolense*. Pathogens of plants (*Phytomonas* spp.) and invertebrates (e.g., *Crithidia* spp. and *Lotmaria* spp.) are also present in this family 20.

Kinetoplastid organisms display some particular features regarding their cellular structure, particularly the presence of a dense mass of mitochondrial DNA (the kinetoplast), and genetic organization, with constitutive transcription of genes contained within polycistronic units and mRNA processing by *trans*-splicing 6. Remarkably, although histone variants and histone post-translational modifications determine regions of start/end of transcription, the control of gene expression in kinetoplastids relies heavily on post-transcriptional mechanisms, on which PRMTs play a significant regulatory role. Moreover, arginine methylation has been identified in trypanosomatid histones 21–23 and, as in other eukaryotes, is likely to be involved in gene expression regulation.

**Figure 2:**
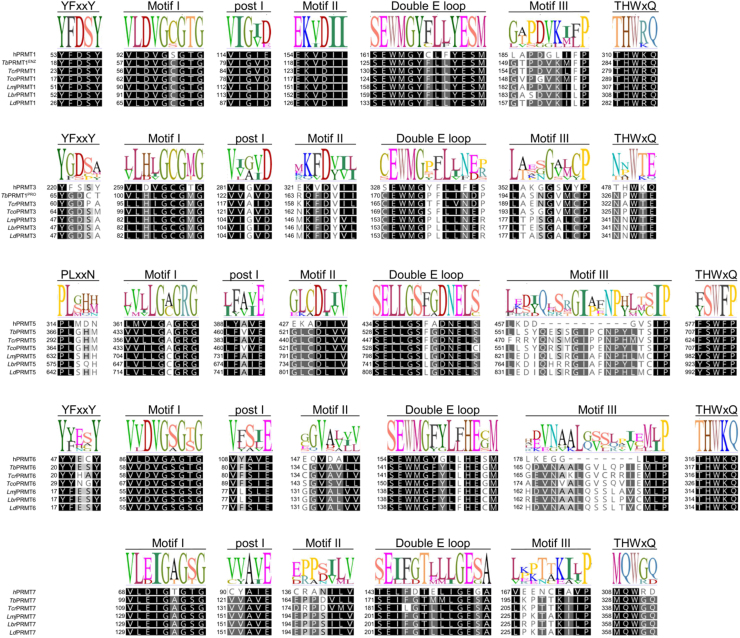
Global alignment of PRMT amino acid sequences from human and trypanosomatids highlighting conserved motifs. Sequences from *Homo sapiens* (h), *T. brucei* (*Tb*), *T. cruzi* (*Tcr*), *T. congolense* (*Tco*), *L. major* (*Lmj*), *L. braziliensis* (*Lbr*) and *L. donovani* (*Ld*) were aligned using Geneious Prime software v.2025.1.3 with Clustal Omega 26. Sequence IDs are provided in Supplementary Table 1.

These parasites possess five genes encoding for canonical PRMTs that are orthologues of the human PRMTs 1, 3, 5, 6 and 7 (Figure 2). The several studies performed on these enzymes have proposed that (1) PRMT1 forms a heterocomplex with the inactive PRMT3 to create the most catalytically active type I PRMT in trypanosomatids, (2) PRMT5 is the only type II PRMT present in trypanosomatids, (3) PRMT6 is a type I PRMT with a narrow substrate range, and (4) PRMT7 is the only type III PRMT in these parasites 24. It is worth saying that the potential existence of a non-canonical type II PRMT in *Leishmania braziliensis* has been recently proposed 25, but it still awaits identification and functional characterisation.

Nevertheless, it has been shown that the impairment of PRMT functions in *Trypanosoma* and *Leishmania* parasites can have profound biological effects, which allows us to propose this group of enzymes as potential drug targets against the diseases caused by these organisms. Furthermore, identifying potent inhibitors for trypanosomatid PRMTs could extrapolate the medical sphere and positively impact agriculture by their applicability on the treatment of crop infections by *Phytomonas* spp. 27, and on the preservation of bee colonies from infections by *Crithidia* spp. and *Lotmaria* spp. 28, which in turn benefits pollination (and, by extension, fruticulture) and honey production.

###  Trypanosoma

All five *T. brucei* PRMTs have been substantially studied, and functional and structural differences in comparison to their human counterparts have been disclosed, which would allow the specific targeting of these enzymes. Importantly, the chemotherapy against *T. brucei* infections in humans and livestock is largely based on eight drugs or drug combinations (Pentamidine, Suramin, Diminazene, Isometamidium, Fexinidazole, Melarsoprol, Eflornithine, and Eflornithine + Nifurtimox) that suffer from high toxicity and recurring appearance of resistant parasites, which reinforces the need for the constant pursue of safer and more effective therapies 29, 30. This continuous development is of particular importance for Chagas’ disease, as the chemotherapy against *T. cruzi* is based on only two highly toxic drugs (benznidazole and nifurtimox) that are largely inefficient during the chronic phase of the disease. Although no study has been performed in any *Trypanosoma* species other than *T. brucei* so far, arginine methylation in both histone and non-histone proteins have been mapped in *T. cruzi* 21, 31, and different from what is observed in humans with methylated arginines generally occurring within arginine-glycine rich motifs, in *T. cruzi* methylated arginines seem to be generally preceded by a cysteine 31, potentially indicating relevant differences in substrate binding between human and *T. cruzi* PRMTs.

It has been shown that the knockout (KO) (but not the knockdown) of *Tb*PRMT1 reduced the growth of bloodstream forms (BSF) *in vitro* and virulence in mice 32, 33. Interestingly, whereas the human PRMT1 usually forms homo-oligomeric structures, the *T. brucei* counterpart forms a ring-like heterodimer with *Tb*PRMT3, and two heterodimers are symmetrically arranged to form the heterotetrameric active enzymatic unit of *Tb*PRMT1. Tetramerization is mostly mediated by van der Waals interactions 34, which likely indicates a dynamic rate of formation and dissolution of this heterotetramer. Thus, although the heterotetrameric profile of *Tb*PRMT1 presents a valuable difference in comparison to the mammalian enzyme, it seems unlikely that a chemical probe would be able to covalently bind to this region and impede tetramerization for long enough to impact parasite fitness. All the same, if achievable, interfering with tetramerization of *Tb*PRMT1 should be a safe approach, as it is unlikely to target the host enzyme.

*Tb*PRMT3 lacks double-E and THW conserved domains (Figure 2), which renders this protein catalytically inactive. Nonetheless, it has been shown that *Tb*PRMT1 and *Tb*PRMT3 are necessary for the stability of each other, and the knockdown of either of them leads to an accumulation of intracellular MMA and decrease in ADMA 32, 35. Thus, given the prozyme profile of *Tb*PRMT3 within this protein complex, *Tb*PRMT1 and *Tb*PRMT3 have been renamed to *Tb*PRMT1 ${}^{\mathrm{ENZ}}$ and *Tb*PRMT1 ${}^{\mathrm{PRO}}$ , respectively 36. Yet, although catalytically inactive, *Tb*PRMT1 ${}^{\mathrm{PRO}}$ is essential for *Tb*PRMT1 activity as it seems to play a significant role in substrate recognition and binding: heterotetramers containing a truncated *Tb*PRMT1 ${}^{\mathrm{PRO}}$ that lacks amino acids 41–52 are unable to bind substrate correctly and, thus, are inactive 34. Noteworthy, the N-terminal region of *Tb*PRMT1 ${}^{\mathrm{PRO}}$ highly diverges from its human counterpart, and might become a potential target for chemotherapy.

It is also noteworthy saying that the KO of *Tb*PRMT1 ${}^{\mathrm{ENZ}}$ caused a large remodelling in the proteome of *T. brucei* BSF, particularly regarding energy metabolism pathways, with a reduction in the levels of glycolytic enzymes and a prominent increase in enzymes participating in proline degradation and in the tricarboxylic acid cycle 33; these pathways are normally utilized by procyclic forms (PCF) to cope with the small amounts of glucose in its natural environment, the gut of tsetse flies. Adaptations in the proteome to fit a procyclic-like metabolism were also noted in *T. brucei* BSF exposed to suramin. Remarkably, *Tb*PRMT1 ${}^{\mathrm{ENZ}}$ and *Tb*PRMT1 ${}^{\mathrm{PRO}}$ were only minorly underexpressed in suramin-exposed cells 37. Thus, it is possible to speculate that a *Tb*PRMT1 inhibitor would have an additive/synergistic effect with suramin, which would allow a reduction in the dose, and therefore in the toxicity, of this drug. Yet, this hypothesis would still require experimental evidence.

**Figure 3:**
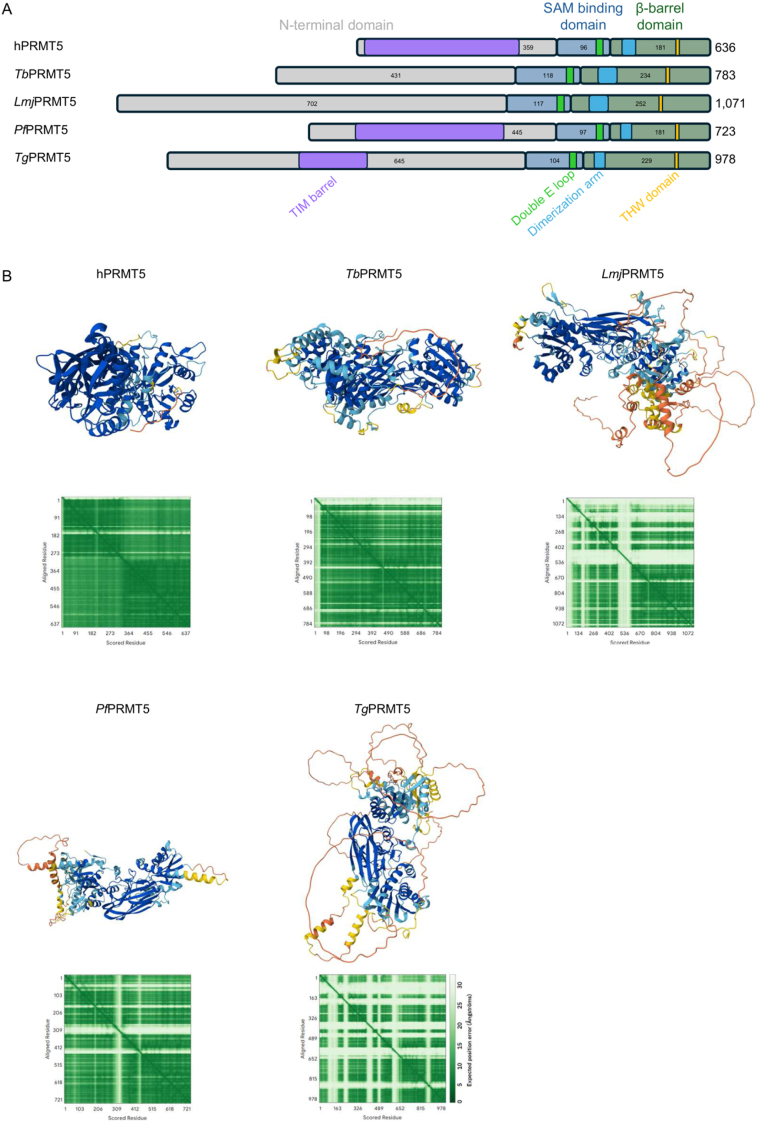
Comparison of domain disposition and predicted structure of PRMT5 in humans and in trypanosomatid and apicomplexan parasites. **(A)** Schematic representation of motifs and relevant regions of PRMT5. N-terminal domains are shown with grey boxes, SAM-binding domains are shown in dark blue, and β -barrel domains are shown in dark green. Numbers within the boxes show the size of each domain. Regions forming a TIM-barrel (purple) and dimerization arms (light blue), as well as substrate binding regions (double E loop (light green) and THW motif (yellow)) are displayed. **(B)** AlphaFold2-predicted structure of PRMT5 in *H. sapiens* (h), *T. brucei* (*Tb*), *L. major* (*Lmj*), *P. falciparum* (*Pf*), and *T. gondii* (*Tg*). Sequences were obtained from Genbank, TritrypDB 44, PlasmoDB 45, or ToxoDB 46 and submitted to AlphaFold 47 server for structure prediction. Predicted Aligned Error plots are shown below each structure.

In addition, diamidine drugs as pentamidine, isometamidium and diminazene aceturate have been shown to accumulate within the mitochondria, and decrease in mitochondrial membrane potential is a rather common initial adaptation in drug resistant *T. brucei* and *T. congolense* strains 30, 38, 39. Therefore, it seems reasonable to think that the inhibition of *Tb*PRMT1 activity, with increased mitochondrial activity, would elevate the accumulation of these drugs inside the mitochondrion and potentiate their effect; experimental confirmation of improvement of drug potency when in combination with a trypanosome PRMT1 inhibitor is still required. Yet, at this moment, it seems plausible to speculate that finding a specific inhibitor of *Tb*PRMT1 could be beneficial either as sole chemotherapy or as part of a combined therapy with the currently used drugs, enabling the use of lower doses and/or the shortage of treatment regimens, thus reducing toxicity and improving adhesion to the treatment. Importantly, the elevated veterinary use of isometamidium and diminazene aceturate (associated with poor dosage and low drug quality) in endemic areas for African Animal Trypanosomiasis led to an increase in reports of drug resistance 40, 41, therefore identifying chemotherapeutical agents that (at least) potentiate the currently used drugs is of utmost importance.

Moreover, drugs such as nifurtimox and benznidazole (both used as the frontline treatment of Chagas’ disease; and nifurtimox is used in combination with eflornithine against sleeping sickness) could also benefit from combinations with PRMT-inhibitors: the trypanosome PRMT5 has been immunoprecipitated associated with tryparedoxin peroxidase 42, a key enzyme in the redox metabolism of trypanosomes 43, and might play a role in regulating enzyme functionality. Although the influence of (potential) arginine methylation on tryparedoxin peroxidase remains to be tested, a dysfunctionality caused by the loss of PRMT5 activity could perhaps potentiate the effect of nifurtimox and benznidazole, given both these drugs act by increasing oxidative and nitrosative stress in the parasites.

Importantly, *Tb*PRMT5 is the only type II enzyme expressed in this parasite, as well as the largest *T. brucei* PRMT, yet the least studied of them and the only one whose structure has not been formally solved. Nevertheless, *Tb*PRMT5 displays peculiar features: it possesses an elongated N-terminal domain which does not contain any known domains (Figure 3**A**), and unlikely the mammalian PRMT5, it does not require any co-factor for its activity 42. Comprehending the molecular divergences that allow *Tb*PRMT5 to function without the presence of any associated protein, and further understanding the role played by the extended N-terminal domain of *Tb*PRMT5 might enable the development of specific inhibitors.

As for *Tb*PRMT6, the disclosure of its structure revealed the presence of some interesting features: the presence of four insert sequences (which are conserved across species of trypanosomatids), a truncated C-terminus, and an active site that is remodelled upon S-adenosylhomocystein (SAH) binding to allow correct arginine substrate orientation. Importantly, the remodelling of the active site involves residues that are conserved in type I PRMTs and might be a common feature of these proteins 48, and thus it seems unlikely that blocking either SAM binding or the structural rearrangement thereafter constitute valuable drug targets. Nonetheless, it has been shown that the knockdown of *Tb*PRMT6 compromises cytokinesis and reduces parasite multiplication (of both BSF and PCF) *in vitro* 49, however further studies are still necessary to confirm whether the knockdown (or KO) of *Tb*PRMT6 would also impact parasite fitness *in vivo* and elevate this enzyme to the position of an actual drug target in *T. brucei*.

Finally, TbPRMT7 seems to be the least promising druggable *T. brucei* PRMT, given (1) its knockdown did not seem to cause any loss of fitness *in vitro* and (2) the loss of its ‘monomethylation only’ function could be rescued by the activity of other PRMTs, particularly *Tb*PRMT1 35.

###  Leishmania

To date, five canonical PRMT encoding genes have been identified in the *Leishmania* genomes. While only a single comprehensive study has been recently accomplished in *Leishmania braziliensis*, most of the efforts have been directed to the characterisation of *Leishmania major* PRMT7, particularly regarding its role in the control of gene expression and in the *Leishmania*-host interaction.

Similar to *T. brucei*, *Lbr*PRMT1 and *Lbr*PRMT3 seem to act in conjunction to form the most active type I PRMT in *L. braziliensis*, and the KO of either protein causes an intracellular accumulation of MMA and a diminution in ADMA 25. One important difference, however, relies on the conservation of the double E loop in *Lbr*PRMT3 (Figure 2), which opens the possibility of this enzyme being somewhat active per se, but given the lack of conservation observed for the THW domain, this will still require functional confirmation. Moreover, the mechanism by which *Lbr*PRMT1 and *Lbr*PRMT3 may interact and the arrangement of this potential quaternary structure is still unknown, and hence precludes any speculation on its druggability.

Nevertheless, even if *Lbr*PRMT1 and *Lbr*PRMT3 form a heterocomplex, it seems likely that they also perform different functions as isolated proteins: the KO of *Lbr*PRMT1, but not of *Lbr*PRMT3, drastically reduced the percentage of infected cells as well as the number of amastigotes per cell in *in vitro* infection assays 25. Yet, we observed that the KO of either protein impaired *L. braziliensis* amastigogenesis *in vitro* 25, possibly because the variety of cues that trigger intracellular parasite differentiation act via pathway(s) independent of *Lbr*PRMT3 activity. Alternatively, it is possible that, in the absence of *Lbr*PRMT3, *Lbr*PRMT1 still retains a basal level of activity that is sufficient to trigger differentiation inside the macrophage, or it is also possible that *Lbr*PRMT1 performs moonlighting functions necessary for intracellular amastigote formation, which are independent of *Lbr*PRMT3. Thus, it seems that inhibiting *Lbr*PRMT1 itself would be a more attractive strategy than trying to impede a potential *Lbr*PRMT1:*Lbr*PRMT3 dimerization.

Concordantly with the need of the major type I PRMT for efficient cell infection and amastigote survival in *L. braziliensis*, the only known type II PRMT expressed in *L. braziliensis* (*Lbr*PRMT5) is also required for efficient cell infection and amastigote multiplication *in vitro*. Moreover, although the pattern of proteins harbouring symmetrically dimethylated arginine residues was slightly altered upon the KO of *Lbr*PRMT5 25, there was no major reduction in the intracellular levels of SDMA. One important limitation to be highlighted is the potential promiscuity of the anti-SDMA antibodies in extracts of *Leishmania*. Therefore, a more comprehensive investigation on SDMA-harbouring proteins and of *Lbr*PRMT5 interaction partners will be required.

Importantly, *Leishmania* PRMT5 displays a very elongated N-terminal domain that makes it 30% longer than the *T. brucei* orthologue and 60% longer than its human counterpart (Figure 3**A**) and might eventually be specifically targeted by a chemical probe. The structure and the function (if any) of this domain is still to be formally determined, but structural prediction of the *Leishmania major* PRMT5 shows this N-terminal region is highly disordered (Figure 3**B**). Whether this region plays a role in the allosteric regulation of its enzymatic activity or may serve as a scaffold for the activity of other proteins (a scaffolding property of PRMT5 has been recently demonstrated in zebra fish 50) is still to be determined.

Thus, it seems clear that understanding the mechanisms coordinated by the activity of *Lbr*PRMT1 and *Lbr*PRMT5 in the metacyclic and early amastigote stages of *L. braziliensis* is of great importance, as it may reveal the effector proteins that are actually implicated in the loss of parasite fitness at that stage. Moreover, expanding the study to other species of *Leishmania* is of interest, particularly in the face of the different clinical manifestations of the disease.

One example of this is that the KO of PRMT6 or PRMT7 in *L. braziliensis* did not impact *in vitro* cell infection or amastigote differentiation 25, whereas the KO of PRMT7 in *L. major* positively impacted parasite infectivity, both *in vivo* and *in vitro* 51, 52. However, different from what is generally expected, the worsening of infection outcome was not related to increased parasite burden, but to increased neutrophil infiltration 52. Also in *L. major*, it was recently shown that the overexpression on *Lmj*PRMT6 negatively impacts parasite fitness during *in vivo* infection 53. Notably, the increased expression of *Lmj*PRMT6 did not visually alter the pattern of arginine methylation in promastigotes of *L. major*, which indicates *Lmj*PRMT6, likewise *Tb*PRMT6, methylates only a small number of substrates; alternatively, it is possible *Lmj*PRMT6 may perform moonlight functions that impact parasite fitness without altering arginine methylation 53. In the light of this, *Leishmania* PRMT6 and PRMT7 seem to be less attractive drug targets than *Leishmania* PRMT1 and PRMT5. Nevertheless, if possible, finding a chemical probe that could work as an activator of *Leishmania* PRMT6 and/or PRMT7 could perhaps constitute a valuable strategy to help alleviate the pathology associated with the infection, particularly for cutaneous leishmaniasis.

Overwhelmingly, although the pan-methyltransferase inhibitor sinefungin has been shown to display potent activity against *Leishmania* parasites *in vitro* and *in vivo* 54–56, the *Leishmania* PRMTs are not among the main targets of this antimicrobial compound, as revealed by a genome-wide analysis of sinefungin-resistant *Leishmania infantum* promastigotes 57, which opens the possibility of a combined therapy between sinefungin and trypanosomatid-specific PRMT inhibitors, as these are unlikely to compete for the same target, which also makes it harder for cross-resistance to appear.

## APICOMPLEXAN PRMTs

Although not listed among the 20 neglected tropical diseases elected by the World Health Organisation, diseases caused by apicomplexan parasites, such as malaria (*Plasmodium* spp.), toxoplasmosis (*Toxoplasma gondii*) and cryptosporidiosis (*Cryptosporidium* spp.), still impose a huge burden on human populations: for instance, 229 million cases and over 400 thousand deaths by malaria were reported in 2019, mostly due to *Plasmodium falciparum* infections 58, while around 30% of the human population has been infected by *T. gondii* and around 190 thousand cases of congenital toxoplasmosis are reported yearly 59. Moreover, only in 2016, acute cryptosporidiosis caused more than 48 thousand deaths and a loss of approximately 4.2 million disability-adjusted life-years 60.

Different from kinetoplastids, the control of gene expression in apicomplexans follows more closely the general rules for more complex eukaryotes, in which a *cis*-acting region controls the level of transcription of a given gene, which generates a pre-mRNA containing exons and introns, from which the introns are removed by *cis*-splicing 61, 62. Nonetheless, epigenetic mechanisms such as the positioning of histone variants in the genome affect the binding of the preinitiation complex to promoter regions and, thus, the rate of transcription 63. Moreover, although apicomplexan parasites are less dependent on post-trancriptional control than kinetoplastids, the correct processing of the pre-mRNA is still vital for the correct gene expression and protein function, as well as for life stage transitioning 61, 64. In this sense, the methylation of arginine in histones and in proteins involved in mRNA processing in apicomplexan parasites is linked to the control of gene expression, cell division and life cycle progression in these organisms.

**Figure 4:**
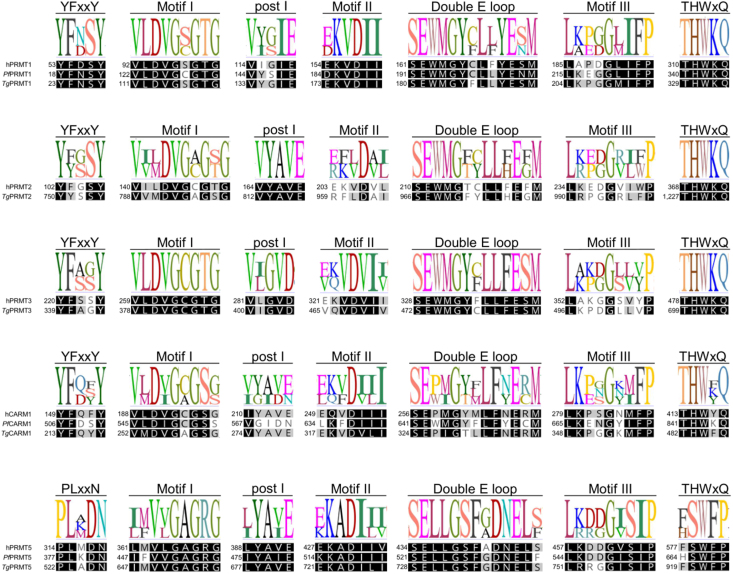
Global alignment of PRMT amino acid sequences from human and apicomplexan parasites highlighting conserved motifs. Sequences from *Homo sapiens* (h), *P. falciparum* (*Pf*), and *T. gondii* (*Tg*) were aligned using Geneious Prime software v.2025.1.3 with Clustal Omega 26. Sequence IDs are provided in Supplementary Table 1.

A variable number of PRMT coding genes is found in apicomplexan parasites: three in *P. falciparum*, four in *Cryptosporidium parvum* (all annotated as hypothetical), and five in *T. gondii*. It is noteworthy that these three parasites possess at least two type I and one type II, but no type III, PRMTs. Moreover, different from trypanosomatids, orthologues of the human PRMT4/CARM1 and PRMT2 enzymes are found in this group (Figure 4). Even though the research on the biological relevance of apicomplexan PRMTs is still limited, they have been shown to be involved in core cellular mechanisms, from gene expression to cell division, and might rise as potential drug targets in the future. Additionally, the identification of new suitable drug targets in Apicomplexa could also benefit advances in the treatment of diseases of medical and veterinary relevance caused by *Cyclospora* sp., *Babesia* sp., and *Theileria* spp. As for the latter, a screen of epigenetic drugs against *T. annulata* recently showed that BVT-948 and TCE-5003 (both of which can inhibit mammalian PRMTs) 65, 66 display antiparasitic effect in *in vitro* assays; molecular docking analyses further showed that both molecules might bind a *T. annulata* PRMT 67.

###  Plasmodium

Several *Plasmodium* species cause malaria around the world, yet all the studies on *Plasmodium* PRMTs have been carried out in *P. falciparum*, which causes the most severe form of the disease in humans. In this species, a total of 843 proteins, representing more than 16% of the *P. falciparum* proteome, were found to carry methylated arginine residues distributed among ring (486), trophozoite (536) and schizont (367) stages, 170 of which were shared between the three studied stages 68.

Additionally, a recent comprehensive and quantitative proteomic analysis identified methylated arginine residues in the histones H3 and H3.3 in trophozoites and (mature and immature) gametocytes of *P. falciparum* 69. Pertinently, distinct patterns of distribution, abundance and PTM combinations that included arginine methylation were observed for each biological form analysed and were particularly prominent in gametocytes. For example, arginine 8 was largely unmodified in both H3 and H3.3 in trophozoites (although symmetric and asymmetric H3R8 methylation can be detected by western blotting), but its mono- and dimethylation increased in gametocyte forms. Moreover, the levels of dimethylated H3R17 and monomethylated H3R40 increased significantly from trophozoites to immature gametocytes 69–71.

It is noteworthy, however, that beyond the change in abundance, the same arginine methylation was involved in different histone PTM cross talk networks across life cycle stages, for instance, whereas dimethylated H3R17 correlates mostly negatively with the presence of other PTMs in trophozoites, its presence strongly positively associates with the existence of other PTMs in both mature and immature gametocytes. Strikingly, dimethylated H3R17 is the most connected PTM in H3 of mature gametocytes, and alongside acetylated K18 and K23 residues, forms a triad of gene expression activation markers required in mature gametocytes. Concordantly, dimethylated H3R17 was found to be depleted in heterochromatic regions in both ring and gametocyte stages 70. Moreover, H3R17 displays a dynamic localization over the genome: while it is mostly found in intergenic or in inter/intragenic intersection regions in ring stages, it becomes more prevalent in intragenic regions in gametocytes 70. These observations confirm that methylated arginine residues partake in a complex, dynamic and combinatorial histone code that is involved in proliferation and differentiation processes in *P. falciparum* 69. Concordantly, methylated arginine residues have also been found in members of protein superfamilies involved in antigenic variation in *P. falciparum* 68, which implies a potential role of PRMTs activity in the maintenance and progression of *Plasmodium* infection.

Yet, despite the ubiquitous presence of methylated arginine in *P. falciparum*, only *Pf*PRMT1 has been biochemically characterised and shown to display a canonical type I activity, as it was able to catalyse the formation of MMAs and ADMAs. Moreover, recombinant *Pf*PRMT1 was able to methylate several proteins involved in RNA metabolism, as well as histones *in vitro*, implying an important role in the control of gene expression in this parasite 72. This correlates with the wide intracellular distribution of *Pf*PRMT1 in ring, trophozoite and schizont forms, although displaying a clear concentration in the nucleus, particularly in schizonts 72.

*Pf*PRMT5, the only type II PRMT expressed by *P. falciparum*, also displays a wide distribution in asexual stages of the parasite and was detected in both cytoplasmic and nucleic fractions 71, 73. The ability of *Pf*PRMT5 to methylate histones, particularly at H3R2, has been confirmed *in vitro* and *in vivo* 71. The loss of *Pf*PRMT5 affects the global transcriptome of intraerythrocytic stages of *P. falciparum*, particularly at the late stages of intracellular development and mostly affecting the expression of genes related to cell invasion and motility. Moreover, *Pf*PRMT5 likely influences gene expression by mediating the correct assembly of *P. falciparum* spliceosome: the use of the PRMT inhibitor adenosine periodate during *in vitro* translation of spliceosome component *Pf*SmD1 negatively impacted its interaction with two other spliceosome components (*Pf*SMN and *Pf*Tu-TSN). Immunoprecipitation of *Pf*SmD1 revealed its association with *Pf*PRMT5 *in vivo* 73. It is worth noting that human PRMT5 is also known to influence the spliceosome assembly, and the inhibition of type II human PRMTs resulted in intron retention 74. In *P. falciparum*, absence of *Pf*PRMT5 caused a large increase in alternative splicing events, particularly exon skipping and mostly affecting genes related to virulence 71.

The disruption of *Pf*PRMT5 caused a reduction in the number of merozoites formed and in the infection capacity of these merozoites 71. This phenomenon seems to be linked at least partially to the lack of H3R2 (di-)methylation at the UTRs of genes found downregulated in *Pf*PRMT5-KO parasites and due to the interaction between *Pf*PRMT5 and transcription factors such as *Pf*AP2-I, *Pf*BDP1 and *Pf*GCN5 71. Of note, the latter is an important regulator of stress response in *P. falciparum*, and its inhibition sensitises parasites to artemisinin 75. It is not clear at the moment whether the inhibition of *Pf*PRMT5 could affect the activity of *Pf*GCN5, which could perhaps potentiate the action of artemisinin. Nonetheless, a small screen of PRMT5 inhibitors has recently shown that the inhibition of *Pf*PRMT5 by Onametostat can affect parasite viability and reduce the rate of red blood cell invasion 76. The importance of *Pf*PRMT5 during the invasion red blood cells suggest this is a relevant drug target in *Plasmodium*, and the development of more selective inhibitors are of interest. Of note, *Pf*PRMT5, likewise its counterpart in humans, contains a TIM barrel in its N-terminal domain (Figure 3), which implies it might need to interact with another macromolecule (which may perhaps function as a co-factor) for its activity. In humans, the interaction of hPRMT5 with its co-factor MEP50 is being exploited as a potential target against cancer 13, 77, 78. Likewise, potential differences in the interaction of *Pf*PRMT5 with another protein versus hPRMT5 and MEP50 might be exploited as a suitable drug target.

Different from *Pf*PRMT1 and *Pf*PRMT5, no studies have focused on the characterisation of *Pf*PRMT4/CARM1, and, as such, its function remains elusive. Nevertheless, *Pf*PRMT4/CARM1 seems to be the only essential PRMT in the parasite 79. Concordantly, ellagic acid, that is known to inhibit the methylation of R17 in histone H3 by PRMT4/CARM1 in human cells 80, was active against asexual and early gametocytes of *P. falciparum* 81, whereas the pan-methyltransferase inhibitor sinefungin was mostly effective against asexual parasites *in vitro*. Noteworthy, both ellagic acid and sinefungin were active against two multi-drug-resistant strains of *P. falciparum* 81.

Moreover, it has been shown that inhibitors of human PRMT4/CARM1 are able to inhibit intraerythrocytic parasite proliferation: 1-benzyl-3,5-bis-(3-bromo-4-hydroxybenzylidene)piperidin-4-one, that inhibited 50% of human PRMT4/CARM1 activity at 8.6 μ M 82, completely eliminated the intracellular growth of *P. falciparum* at 10 μ M 83, and a 3,5-bis-(3-bromo-4-hydroxybenzylidene)thiopyran-4-one that was largely inactive against human arginine and lysine methyltransferases (up to a concentration of 100 μ M) 84 displayed a half maximum effective concentration (EC ${}_{50}$ ) against intra-erythrocytic forms of *P. falciparum* at less than 1 μ M and virtually abolished parasite proliferation at 10 μ M 83. Whether these molecules, as well as ellagic acid, actually target *Pf*PRMT4/CARM1 is currently unknown, but these initial results do encourage the pursuit of PRMT inhibitors as a new avenue in the fight against malaria. Moreover, assuming that ellagic acid inhibits *Pf*PRMT4/CARM1 activity and that this enzyme is responsible for the methylation of H3R17 in *P. falciparum* 69, it is plausible that the inhibition of *Pf*PRMT4/CARM1 precludes/disturbs the deposition of other epigenetic marks on H3 which, in turn, disconcerts the progression of trophozoites into transmissible life stages.

Yet, whether blocking *Plasmodium* PRMT activities would impair the parasite’s antigenic variation and consequently the escape from the immune response and/or block its differentiation and progression in the intraerythrocytic life cycle or the transmission to the vector is still to be evaluated. On that matter, the genetic manipulation, particularly the KO, of *Plasmodium* PRMTs would be an important step towards understanding the relevance of these enzymes in the viability, metabolic regulation, cell cycle progression and cell infection capacity of malaria parasites.

As for the moment, in addition to the development of novel, *Plasmodium*-specific PRMT inhibitors, the repurposing of existing PRMT inhibitors with limited activity against the human enzymes may present a new opportunity in the fight against malaria, particularly if these molecular entities can be effective against the panel of *Plasmodium* species that cause human malaria, along with testing their potential against drug-resistant *Plasmodium* strains, and the potential combination of these molecules with the currently used antimalarial drugs are important steps for the development of new chemotherapeutical schemes against *Plasmodium* infections, particularly given the rise of drug resistance in Africa 85.

###  Toxoplasma

Despite having four genes encoding for type I (orthologues of PRMT1, PRMT2, PRMT3, and CARM1) and one encoding for a type II PRMT, which ultimately generate ADMA and SDMA, respectively, only MMA sites have been mapped in *T. gondii* tachyzoites so far. Whereas this clearly does not comprise the whole complexity of arginine methylation in *Toxoplasma*, it showed that this PTM is present in almost 4% of the total *T. gondii* proteome, being intimately involved in a wide variety of metabolic processes, but particularly with nucleic acid metabolism: of the 309 proteins identified harbouring MMA, 207 could have their function predicted, and 34% of these were likely involved in DNA or RNA metabolism 86. Notably, approximately 90% of the MMA proteins identified also harboured phosphorylated residues, indicating that a dynamic crosstalk between PTMs likely happens in *T. gondii* 86.

Importantly, many DEAD box helicases, splicing and transcription factors have been identified harbouring MMA, including apicomplexan Apetala2 transcription factor orthologues, which are thought to be involved in life cycle stage transitions 86, 87. Indeed, a large modification in the transcriptome, and by extension in gene expression, occurs during *T. gondii* tachyzoite-bradyzoite transition 88, and the positioning and post-translational modification of histones play an important role in the process by allowing access to different regions of the genome 4. Particularly regarding arginine methylation, at least three *T. gondii* PRMTs (*Tg*PRMT1, *Tg*PRMT4/CARM1 and *Tg*PRMT5) have histones as substrates 89–91 and marks for mono- and dimethylated arginines have been found in H3 and H4 91, among which the dimethylation of H3R17 has been proposed as a marker for active promoters in both tachyzoites and bradyzoites 89, although this seems to be restricted to a subset of promoters rather than a global mark for active transcription 92. Of note, dimethylated H3R17 colocalizes with *Tg*PRMT4/CARM1 in the genome 89.

Interestingly, the treatment of tachyzoites with an inhibitor of *Tg*PRMT4/CARM1 prior to cell infection induced cyst formation after two to three rounds of intracellular parasite replication 89. Hence, the inhibition of *Tg*PRMT4/CARM1 might become a potential chemotherapeutical strategy against the reactivation of the acute phase, which often causes encephalitis and/or retinochoroiditis, by limiting tachyzoite multiplication in immunocompromised patients and enforce re-encystation.

Moreover, impairing tachyzoite multiplication is also a suitable strategy to control the pathogenesis of toxoplasmosis, particularly if the infection occurs during pregnancy, which often incurs in vertical transmission to the foetus and results in miscarriages or in birth defects such as encephalitis, hydrocephalus, retinochoroiditis, among others 93. The genetic KO of *Tg*PRMT1 generated parasites that multiply slower intracellularly, form smaller plaques, are more susceptible to osmotic and alkaline stress, and are less virulent to mice mostly due their inability to undergo correct cyto- and karyokinesis 94–96, likely due to a role for *Tg*PRMT1 in the assembly and/or the function of (some) centrosome components. Yet, even though *Tg*PRMT1 accumulates in centrosomal and pericentrosomal regions of intracellular parasites, it is mostly cytoplasmic in tachyzoites 94 and, curiously, mainly methylates nuclear proteins, at least to the level of monomethylation 86, which suggests that these substrates are methylated prior to the import to the nucleus.

Furthermore, several nucleic acid binding proteins were found to be high confidence *Tg*PRMT1 substrates 86, implying a direct role of this enzyme in regulating the control of gene expression in *T. gondii*. *Tg*PRMT1 has been co-immunoprecipitated with and shown to methylate the RNA silencing machinery component *Tg*Argonaute 95, 97, as well as has been shown to methylate the C-terminus of *Tg*SsossB, a region that is important for its interaction with the RNA-binding protein *Tg*Alba2 and potentially with one isoform of *Tg*Alba1, both of which are intimately involved in gene expression regulation in the parasite 86, 96, 98. Whether the methylation of the RGG domain of *Tg*SsossB might influence its interaction with Alba proteins is still to be assessed, but if it does, it is possible to hypothesize that the chemical inhibitions of *Tg*PRMT1 would indirectly impact Alba protein function, and by extension affect the control of gene expression in the parasite.

Interestingly, *Tg*PRMT1 methylates itself 86, which may imply that it controls its own activity, at least under specific circumstances, but the relevance of this auto-methylation has not been assessed yet. Moreover, differently from *T. brucei* and *L. braziliensis*, *T. gondii* PRMT1-KO cells display a decrease in the intracellular levels of both MMA and ADMA, with no clear alteration in the levels of SDMA 86, which suggests that type I and type II *T. gondii* PRMTs have, at most, a minor overlap in functions that cannot be assessed visually. Determining the extent of the protein methylation by the *T. gondii* type II PRMT will be detrimental to understanding of the interplay between PRMTs in this parasite.

*T. gondii* also expresses an orthologue of PRMT5. Different from its human orthologue and its counterpart in *Plasmodium*, *Tg*PRMT5 displays a highly elongated and disordered N-terminal region, albeit retaining a TIM barrel structure (Figure 3**A**and **B**). Whether this region is important for the allosteric regulation of the enzyme or serves as a platform for other reactions is still to be studied, as is the role played by the TIM barrel in the enzymatic activity of *Tg*PRMT5. Nonetheless, *Tg*PRMT5 is active and can mono- and symmetrically dimethylate histones. Histone methylation by *Tg*PRMT5 is likely more important in bradyzoite forms when this enzyme displays a dual nuclear-cytoplasmic localisation. *Tg*PRMT5 is only found in the cytoplasm of tachyzoite forms 90.

The genetic KO of *Tg*PRMT5 should allow a better comprehension of its role in the tachyzoite-bradyzoite transition and in the intracellular survival of bradyzoite forms. This would also help to reveal the extent of arginine methylation mediated by *Tg*PRMT5, as well as the interplay and (lack of) intersection between type I and type II *T. gondii* PRMT activities. In the hypothesis that *Tg*PRMT5 activity is necessary for the survival of bradyzoites, a combination of *Tg*PRMT4/CARM1 and *Tg*PRMT5 inhibitors might become a potential strategy to eliminate *T. gondii* in chronically infected patients.

## AMOEBA PRMTs

Amoebae are a polyphyletic group of protozoa morphologically related by their ability to generate pseudopods for their motility giving them a characteristic variable and irregular shape. During their life cycle, amoebae are able to alternate between a vegetative and mobile form, called the trophozoite, and a resistant form, called the cyst, in favourable and hostile environmental conditions, respectively. While most amoebae can live autonomously in the environment, thus named free-living amoebae (FLA), some are parasitic, necessitating a host for their development, such as *Entamoeba histolytica*, an enteric pathogen that causes intestinal amebiasis*,* infecting about 100 million people and causing more than 11 thousand deaths annually 99. Moreover, some FLA are qualified as amphizoic, as they are also able to cause severe infections, either at the ocular level, *i.e.* amoebic keratitis for *Acanthamoeba sp.*, or at the cerebral level, *i.e.* granulomatous amoebic encephalitis for *Acanthamoeba sp.*, or *Balamuthia mandrillaris* and primary amoebic meningoencephalitis for *Naegleria fowleri*. While only several hundreds of FLA cerebral infections have been reported so far, with a presumable underestimation of the number of cases, *Acanthamoeba* ocular infections concern mainly lens wearers with a variable incidence worldwide ranging from 0.13 to 33 cases per million people 100, 101. Although metronidazole is the standard therapy for intestinal amoebiasis despite some adverse effects and few cases of resistance 102, no consensus treatment has been established for FLA cerebral infections, empirically associating a large diversity of drugs, and the current therapy for amoebic keratitis, associating chlorhexidine or polyhexamethylene biguanide with a diamidine (propamidine), is long-lasting, for up to one year, with recurrence in 10% of the cases 103, 104.

In the genome of *E. histolytica*, 4 type I and 1 type II PRMT genes have been identified. The type I *Eh*PRMTs display the highest homology with the human PRMT1, with 42%, 46% and 48% similarity for *Eh*PRMT1a, *Eh*PRMT1b and *Eh*PRMT1c, respectively, while the type II *Eh*PRMT is related to human PRMT5 with 44% similarity. Also, an atypical PRMT1 orthologue (*Eh*PRMT-A), which shares only 32% similarity to hPRMT1, has been identified. All type I *Eh*PRMTs are expressed in the trophozoite form, but it has been described that the abundance of *Eh*PRMT1c transcripts decreased substantially just after intestinal infection in mice, suggesting a role of this isoform in adaption to different environments 105, 106.

**Figure 5:**
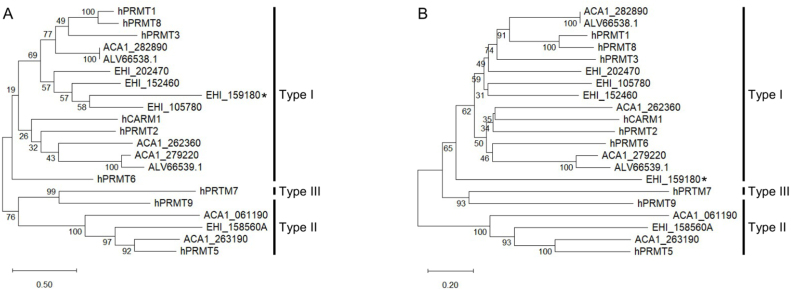
Phylogenetic trees constructed for human, *E. histolytica*, and *A. castellanii* PRMT protein sequences. Sequences were retrieved from Genbank or AmoebaDB 113 and aligned using Clustal Omega 26 and submitted to Maximum-likelihood **(A)** and Neighbour-joining **(B)** phylogenetic analyses in MEGA12 114 with 500 bootstraps. Sequences IDs are provided in Supplementary Table 1. ALV66538.1 and ACA1_282890 display the same sequence. Asterisk indicates *Eh*PRMT-A.

All type I *Eh*PRMTs were predicted to have a S-adenosyl-methionine-binding domain (AMBD) and a barrel-like structure domain (BLD) containing the active site and the dimerization domain, as for other PRMTs 106. The recombinant *Eh*PRMT1a was shown to form homodimers and homotetramers and to exhibit methyltransferase activity towards histones 106. Moreover, immunofluorescence analyses showed that histone methylation marks associated with transcriptional repression are distributed throughout the *E. histolytica* nucleus and that *Eh*PRMT1 proteins are colocalized with H4 histone in the trophozoite nuclei 106, 107.

The atypical *Eh*PRMT-A, despite lacking several canonical residues of type I PRMTs, has shown the ability to methylate histones *in vitro* 108. This enzyme has also been reported to associate with the transcription factor *Eh*TSN and is therefore likely involved in gene transcription regulation 109. Curiously, however, *Eh*PRMT-A is localised in the cytoplasm and in the perinuclear region, with an increase in expression and an apparent partial change in localisation towards the nucleus during heat shock 108. *Eh*PRMT-A was also found to increase in expression and migrate to the cell periphery during red blood cell phagocytosis, perhaps suggesting a role in remodelling the cytoskeleton during phagocytosis. Concordantly, the knockdown of *Eh*PRMT-A caused a reduction in cell migration. However, cells knocked down for *Eh*PRMT-A showed increased erythrophagocytosis and multiplied faster *in vitro*. Therefore, despite the potential interest in *Eh*PRMT-A as a potential drug target due to its sequence and structure divergence in relation to the human PRMTs, the potential increase in parasite multiplication and in red blood cell phagocytosis caused by *Eh*PRMT-A inhibition indicate this may not be an adequate target against *E. histolytica*.

*Entamoeba* species also present one type II PRMT. In *Entamoeba invadens*, a species for which highly efficient *in vitro* encystation protocols have been established, SDMA-harbouring proteins are observed in both trophozoites and cysts, with levels increasing and displaying a dynamic intracellular localisation during encystation 110. Treatment with EPZ015666, an inhibitor of mammalian PRMT5, decreased the levels of SDMA in trophozoites and during encystation, indicating it can also bind and inhibit *Ei*PRMT5; the binding of EPZ015666 to *Ei*PRMT5 is supported by molecular docking. Although treatment with EPZ015666 can reduce both trophozoite viability and the number of cysts formed *in vitro*, this is only achieved by concentrations much above those used in mammalian cells 110. Nevertheless, the development of more specific *Entamoeba* PRMT5 inhibitors might constitute a potential future direction in the development of amoebicidal molecules. The reduction in cyst formation through the inhibition of *Entamoeba* PRMT5 might complement the current chemotherapy targeting trophozoite forms, and help reducing parasite transmission.

**Figure 6:**
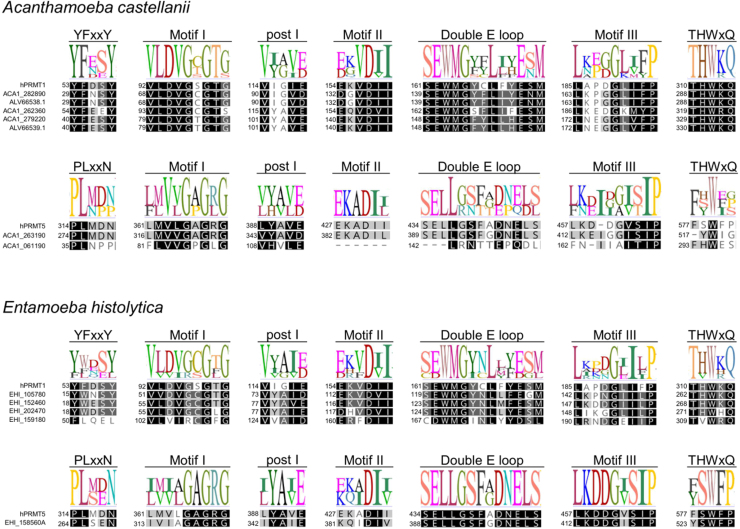
Global alignment of PRMT amino acid sequences from human and amoebas highlighting conserved motifs. Type I PRMT sequences from *A. castellanii* and *E. histolytica* were aligned to the human PRMT1, while sequences for type II enzymes were aligned to the human PRMT5. Sequences were aligned using Geneious Prime software v.2025.1.3 with Clustal Omega 26. Sequence IDs are provided in Supplementary Table 1.

The FLA *Acanthamoeba castellanii* possesses five genes encoding for PRMTs, of which two have been shown to be relevant (but not essential) for encystation 111, 112. Maximum-likelihood and Neighbour-joining phylogenetic trees (Figure 5**A** and **B**, respectively) constructed with PRMT amino acid sequences from *Homo sapiens*, *E. histolytica*, and *A. castellanii* show that three out the five PRMTs found in *A. castellanii* are likely type I enzymes, and two likely correspond to type II PRMTs. Of note, the enzyme characterised by Moon *et al*. (2017) as *Ac*PRMT1 (GenBank: KT345168; protein ID: ALV66538.1) displays the same sequence as the one encoded by the gene ACA1_282890 (AmoebaDB), and that named as *Ac*PRMT5 (GenBank: KT345169; protein ID: ALV66539.1) by Moon *et al*. (2016) is 74% identical to ACA1_279220 (AmoebaDB).

Curiously, however, *Ac*PRMT5 (GenBank: KT345169; protein ID: ALV66539.1) clustered together with other type I PRMTs, including that characterised by Moon *et al*. (2017) as *Ac*PRMT1 (GenBank: KT345168; protein ID: ALV66538.1), which suggests they are both type I PRMTs. An inspection in the amino acid sequence of ALV66539.1 shows it indeed possesses conserved PRMT motifs, including a canonical type I PRMT YFxxY motif (Figure 6). On the other hand, the protein encoded by the gene ACA1_263190 (AmoebaDB) clustered closely to the human PRMT5. The protein encoded by the gene ACA1_061190 (AmoebaDB) also clustered together with other type II PRMT sequences, albeit more distantly: ACA1_061190 appears to lack conserved motif II and double E loop regions (Figure 6), indicating it is either not functional or represents an atypical type II PRMT found in this amoeba. Given that no biochemical characterisation has been performed in any of the *Ac*PRMTs, it is difficult to pinpoint whether they are all functional and whether either one of them is essential for trophozoite or cyst survival. Nevertheless, likewise *Entamoeba*, a PRMT inhibitor that could reduce or eliminate *Acanthamoeba* cyst formation would be of interest in a combined therapy against trophozoites to help reducing transmission. The recent advances in the development of genome editing tools using CRISPR-Cas9 in *E. histolytica* 115 and *A. castellanii* 116 will certainly serve as powerful tools to efficiently and rapidly determine in detail the role of each PRMT in these amoebae.

In the FLA *Naegleria fowleri*, the *Nf*PRMT1 has been defined as a potential drug target by the Seattle Structural Genomics Center of Infectious Diseases (SSGCID) based on its sequence analysis 117. The analysis of *Nf*PRMT1 crystal structure revealed high sequence identity with *Hs*PRMT1 in the ligand pocket, but some differences in side chain orientation were identified specifically in the FLA enzyme as opposed to all 9 hPRMT isoforms, such as a methionine adjacent to the S-adenosine-homocysteine domain, or the absence of two tyrosines in the substrate binding pocket. These differences could be further used to design inhibitors that specifically target PRMT1 in *N. fowleri*.

## OTHER PROTOZOA

The presence of PRMTs in the genomes of various clades of protozoans suggest they have a high biological relevance for these organisms and might constitute drug targets to fight several different diseases. This, however, does not seem to be the case for diplomonads, in which PRMT genes seem to be absent. In the case of *Giardia* spp., a global genomic and proteomic adaption to replace arginine residues that are conventionally methylated by lysine residues was observed, resulting in rewiring of intracellular networks that typically involve arginine methylated proteins 118.

The social amoeba *Dictyostelium discoideum* is an important model organism for the study of cellular processes such as cell development and differentiation, as well as host-pathogen interactions, particularly due to its genetic tractability and conserved cellular processes 119, 120. Four predicted PRMT-encoding sequences were identified in the *D. discoideum* genome, presumably encoding for *Dd*PRMT2 and *Dd*PRMT4/CARM1, besides genes for *Dd*PRMT1 and *Dd*PRMT5 121–123, but the relevance of all of them still awaits investigation. The use of CRISPR/Cas9 approaches 124, 125 to dissect PRMT function in *D. discoideum* will certainly be of interest to better understand the roles played by these enzymes in model eukaryotes, as well as to better understand the evolution of PRMT-coordinated cellular processes.

## CONCLUDING REMARKS

The World Health Organization has set the goal of eliminating or, at least, controlling the transmission of neglected tropical diseases (NTDs) in the affected countries by 2030 19. This, of course, requires better diagnostic, evaluation, and monitoring tools, as well as facilitated access to medicines and medical supplies, and also more efficient treatments. Among the NTDs, sleeping sickness, Chagas’ disease and leishmaniasis are caused by protozoans and put nearly a billion of people under the risk of infection.

One of the biggest hurdles for treating the diseases caused by protozoa is the lack of efficient chemotherapy: available drugs suffer from high toxicity, appearance of resistant parasites, and low efficacy. Thus, improving the current therapies and developing safer and more effective treatments is of significant interest 85, 126. PRMTs are highly explored targets for cancer treatment, with dozens of molecules in clinical trials, and many more developed every year 11–14. It is foreseen that most of these molecules will not progress beyond preclinical tests, and around 90% of those that enter clinical trials may fail at different instances of the process 15, 16.

As such, given their relevant role in development, differentiation and infection processes, parasite PRMTs could be exploited, in addition to other families of enzymes (such as kinases, for instance), as valuable drug targets. Molecules that fail to interact with or inhibit human PRMTs could be explored as antiparasitic agents and be further developed to become more potent and specific against parasites. Commercially available PRMT inhibitors, as those offered as part of large libraries (from ChemDiv, Otava Chemicals, and Selleck Chemicals, for instance), could be a starting point for the identification of leading molecular scaffolds. The continuous study of PRMT functions, molecular structure, and substrate preferences and mode of binding will allow the discovery and selection of specific inhibitors, including by facilitating Artificial Intelligence-guided screening of compound libraries against these targets.

## SUPPLEMENTAL MATERIAL

All supplemental data for this article are available online at http://microbialcell.com/researcharticles/2026a-campagnaro-microbial-cell/.

.

## CONFLICT OF INTEREST

The authors declare no conflict of interest.

## ABBREVIATIONS

ADMA – asymmetric ω -dimethylarginine

BSF – bloodstream form

FLA – free-living amoebae

KO – knockout

MMA – monomethylarginine

NTDs – neglected tropical diseases

PCF – procyclic forms

PRMTs – protein arginine methyltransferases

PTM – post-translational modifications

RBP – RNA-binding proteins

SAM – S-adenosylmethionine

SDMA – symmetric ω -dimethylarginine
