# Supplementary material for: Protein arginine methyltransferases in protozoan parasites: a new path for antiparasitic chemotherapy?
Source: Microb Cell. 2026 Feb 12;13:86–102. doi: 10.15698/mic2026.02.869 (PMC12925635; doi:10.15698/mic2026.02.869)
Supplement: Supplementary file 1 — . [file mic-13-086-s01.pdf]

Supplementary Table 1

Organism	PRMT	Database and GeneID				
		Genbank	TritrypDB	PlasmoDB	ToxoDB	AmoebaDB
Human	1	NP_001527.3				
	2	NP_001526.2				
	3	NP_005779.1				
	4 (CARM1)	NP_954592.1				
	5	NP_006100.2				
	6	NP_060607.2				
	7	NP_001364947.1				
	8	NP_062828.3				
	9	NP_612373.2				
Trypanosoma brucei	1 (PRMT1^{PRO})		Tb927.1.4690			
	3 (PRMT1^{ENZ})		Tb927.10.3560			
	5		Tb927.10.640			
	6		Tb927.5.3960			
	7		Tb927.7.5490			
Trypanosoma cruzi	1		C4B63_41g251-t42_1			
	3		C4B63_23g39-t42_1			
	5		C4B63_43g173-t42_1			
	6		C4B63_60g173-t42_1			
	7		BCY84_16438_t1			
Trypanosoma congolense	1		TcIL3000_1_1890.1			
	3		TcIL3000_10_2970.1			
	5		TcIL3000_10_500.1			
	6		TcIL3000_5_4500.1			
	7		*Incomplete*			
Leishmania major	1		LmjF.12.1270			
	3		LmjF.03.0600			
	5		LmjF.21.1450			
	6		LmjF.16.0030			
	7		LmjF.06.0870			
Leishmania braziliensis	1		LbrM.20.6060			
	3		LbrM.15.1340			
	5		LbrM.21.1830			
	6		LbrM.16.0040			
	7		LbrM.06.0840			
Leishmania donovani	1		LdBPK_120850.1.1			
	3		LdBPK_030580.1.1			
	5		LdBPK_211690.1.1			
	6		LdBPK_160040.1.1			
	7		LdBPK_060900.1.1			
Plasmodium falciparum	1			PF3D7_1426200.1		
	4 (CARM1)			PF3D7_0811500.1		
	5			PF3D7_1361000.1		
Toxoplasma gondii	1				TGME49_219520	
	2				TGME49_500120	
	3				TGME49_252420	
	4 (CARM1)				TGME49_294270	
	5				TGME49_215560	
Entamoeba hystolytica	1a					EHI_105780
	1b					EHI_152460
	1c					EHI_202470
	Atypical					EHI_159180
	5					EHI_158560A
Acanthamoeba castellanii	1	ALV66538.1				ACA1_282890
	-					ACA1_262360
	-					ACA1_279220
	-	ALV66539.1				
	-					ACA1_061190
	-					ACA1_263190
